# Metabolic Profiling and Investigation of the Modulatory Effect of *Fagonia cretica* L. Aerial Parts on Hepatic CYP3A4 and UGT2B7 Enzymes in Streptozotocin—Induced Diabetic Model

**DOI:** 10.3390/antiox12010119

**Published:** 2023-01-03

**Authors:** Shahzad Kamran, Rukhsana Anwar, Afifa Noor, Muhammad Ihsan Ullah, Alaa A. Bagalagel, Mohammed M. Aldurdunji, Saiqa Ishtiaq

**Affiliations:** 1 Punjab University College of Pharmacy, University of the Punjab, Lahore 54000, Pakistan; 2 Centre of Excellence in Molecular Biology, University of the Punjab, Lahore 54000, Pakistan; 3 Department of Pharmacy Practice, Faculty of Pharmacy, King Abdulaziz University, Jeddah 21589, Saudi Arabia; 4 Department of Clinical Pharmacy, College of Pharmacy, Umm Al-Qura University, P.O. Box 13578, Makkah 21955, Saudi Arabia

**Keywords:** *Fagonia cretica*, Zygophyllaceae, UGT2B7, CYP3A4, mRNA expression, molecular docking, public health, drug discovery

## Abstract

Drug-metabolizing enzymes are either boosted or suppressed by diabetes mellitus. This research was designed to explore *Fagonia cretica* L. aerial parts’ impact on CYP3A4 and UGT2B7 activity and their mRNA expression in diabetic rats. *Fagonia cretica* (*F. cretica*) dried powder was sequentially extracted with *n*-hexane, chloroform, ethyl acetate, methanol, and water. The methanol extract and aqueous fraction presented the most significant potential to decrease the concentration of *alpha*-hydroxyl midazolam, with 176.0 ± 0.85 mg/Kg and 182.9 ± 0.99 mg/Kg, respectively, compared to the streptozotocin (STZ)-induced diabetic group, reflecting the inhibition in CYP3A4 activity. The fold change in mRNA expression of CYP3A4 was decreased significantly by the methanol extract, and the aqueous fraction of *F. cretica* estimated by 0.15 ± 0.002 and 0.16 ± 0.001, respectively, compared with the diabetic group. Morphine metabolism was significantly increased in rats treated with *F. cretica* methanol extract and its aqueous fraction, displaying 93.4 ± 0.96 mg/Kg and 96.4 ± 1.27 mg/Kg, respectively, compared with the metabolism of morphine in the diabetic group, which highlights the induction of UGT2B7 activity. The fold change in mRNA expression of UGT2B7 was significantly increased by the methanol extract and the aqueous fraction, estimated at 8.14 ± 0.26 and 7.17 ± 0.23 respectively, compared to the diabetic group. Phytochemical analysis was performed using high-performance liquid chromatography (HPLC), where the methanol extract showed more flavonoids and phenolic compounds compared to the aqueous fraction of *F. cretica.* The obtained results were further consolidated by molecular docking studies, where quercetin showed the best fitting within the active pocket of CYP3A4, followed by gallic acid, displaying free binding energies (∆G) of −30.83 and −23.12 kcal/mol, respectively. Thus, *F. cretica* could serve as a complementary medicine with standard anti-diabetic therapy that can modulate the activity of the drug-metabolizing enzymes.

## 1. Introduction

The liver is the main organ for drug metabolism and biotransformation [1], where one or more enzymes metabolize endogenous or foreign molecules into more hydrophilic moieties that can be expelled, secreted, or exhaled more readily [2]. It is carried out by phase I and phase II enzymes that catalyze lipophilic substance conjugation with glucuronic acid, sulfate, glutathione, and acetate [3].

Diabetes mellitus (DM) is a metabolic disease that affects about 366 million people worldwide, and the number is likely to increase up to 552 million people worldwide by 2030 [4,5]. Hyperglycemia typically exacerbates oxidative stress in diabetics, resulting in an increased expression of phase I drug-metabolizing enzymes with a concomitant reduction in phase II drug-metabolizing enzymes expression [6]. Diabetes changes the half-life and bioavailability of drugs through an overall increase in CYP enzymes due to a significant increase in total hepatic CYP content [7] accompanied by alterations in the CYP isozyme expression [8,9]. Pharmacokinetic drug interactions involving CYP3A4 induction result in increased metabolism, with rapid drug clearance and therapeutic failure [10], whereas inhibition of the drug-metabolizing enzymes elevates drug risks [11].

Furthermore, increased transcription of the mRNA encoding the genes for drug-metabolizing enzymes results in enzyme induction and increased hepatic metabolism [12]. Meanwhile, the pregnane X receptor (PXR; Nrf2), a nuclear receptor superfamily member expressed in the liver, regulates the expression of phase-I and phase-II drug-metabolizing enzymes involved in the clearance of substances from the body [13]. The primary target for activation of PXR is the induction of CYP3A4, an important phase-I oxidative enzyme [14]. It is worth mentioning that the activity, protein expression, and mRNA level of CYP3A4 in STZ-induced diabetic rats were higher than those in normal rats [15].

However, enzyme inhibition occurs when the respective enzyme cannot effectively metabolize its substrate because of interference by another substance. This may occur because the enzyme is either competitively blocked by another substrate or non-competitively blocked by a substance that may be metabolized by another enzyme [16]. UDP-glucuronosyltransferase (UGT) is an enzyme that catalyzes glucuronic acid moiety attachment to different drugs, xenobiotics, and endogenous compounds [17]. UGT regulation is mediated by the nuclear receptor PXR, and ligand interaction with PXR leads to PXR activity inhibition, acting as an antagonist of the PXR receptor. The inhibition of UGT expression and activity leads to a significant decrease in the metabolism of UGT substrates [18]. In the human liver and kidney, diabetes significantly lowers UGT2B7 enzymatic activity, mRNA expression, and protein levels [19].

Moreover, natural products show a wide array of therapeutic potential, constituting cheaper candidates with minimum side effects and greater acceptability by patients all over the globe with respect to synthetic entities [20]. Additionally, natural products commonly exist in food, cosmetics, and industries, relying on their great multipurpose potential [21]. *F. cretica* belongs to the family *Zygophyllaceae* and is widely distributed in tropical, subtropical, as well as warm temperate areas worldwide, particularly in dry areas [22].

All members of the genus *Fagonia* are greatly interesting to pharmacists based upon the preliminary pharmacological studies that illustrated their pronounced therapeutic efficiency versus a wide array of ailments [23], particularly *F. cretica* aerial parts that showed a notable effectiveness in the alleviation of cancer in its early stages [24]. Furthermore, *F. cretica* is an extensively used ethnomedicinal plant that show diuretic, laxative, carminative, emetic, and abortifacient potential [22]. In addition, the plant has shown anti-diabetic, antipyretic, antioxidant, antimicrobial, and anti-hemorrhagic properties [25]. These activities mainly rely upon its richness with secondary metabolites such as flavonoids, saponins, alkaloids, terpenoids, coumarins, tannins and sterols, as revealed from preliminary phytochemical screening [22].

Although *F. cretica* is widely used in traditional medicine, investigation of its impact on hepatic drug-metabolizing enzymes has not been carried out yet. The current study was conducted to investigate the effects of various extracts of *F. cretica* on CYP3A4 and UGT2B7 activity and mRNA expression in STZ-induced diabetic rats that were divided into two groups, the first study of its kind. The first group was used to evaluate the plant’s effect on phase I drug-metabolizing enzyme activity and expression of CYP3A4 and the second group was used to evaluate the plant’s effect on phase II drug-metabolizing enzyme UGT2B7 activity and expression. Ketoconazole and midazolam were used as the CYP3A4 standard inhibitor and substrate, respectively, whereas rifampicin and morphine were used as the UGT2B7 standard inducer and substrate, respectively. An enzyme-linked immunosorbent assay and gas chromatography–mass spectrometry techniques were used to detect and quantify CYP3A4 and UGT2B7 substrates in liver homogenates, respectively. The mRNA expression of CYP3A4 and UGT2B7 was determined in the liver by using a real-time PCR assay. In addition, the major phytochemicals present in *F. cretica* extracts were identified using the high-performance liquid chromatography (HPLC) analytical technique in gradient mode. A molecular docking study of these secondary metabolites within the active sites of CYP3A4 was performed to consolidate the obtained results.

## 2. Materials and Methods

### 2.1. Plant Materials

The aerial parts of *F. cretica* (Zygophyllaceae) were collected from the Shah-Dab colony area of Lahore. Dr. Zaheer-Ud-Din, Professor of Botany at Government College University in Lahore, identified the plant and issued voucher number (GC. Herb. Bot. 3593). The sample of this plant was deposited in the Government College University herbarium for future reference.

### 2.2. Preparation of Plant Extracts

The extraction was performed progressively where 1 kg of a fine powder of aerial parts of the plant was macerated in methanol (1:5). After 7 days of soaking, the material was filtered through Whatman 1 filter paper, and the filtrate was vacuum-concentrated using a rotary evaporator at 45 °C. The methanol residue was partitioned by *n*-hexane, chloroform, ethyl-acetate, and distilled water for 7 days, and the respective filtrates were vacuum-concentrated using a rotary evaporator. All fractions were dried at room temperature and stored at −20 °C until used. The percentage yields of methanol, *n*-hexane, chloroform, ethyl acetate, and aqueous extracts were 7.1%, 2.35%, 2.86%, 3.98%, and 5.2%, respectively.

### 2.3. Chemicals and Drugs

Lipomed standards of midazolam, *alpha*-hydroxy midazolam, temazepam, morphine, des-alkyl flurazepam, and nalorphine were used in the study. Rifampicin and ketoconazole were arranged through Pacific and Atco Pharma. MSTFA (N-methyl-N-trimethylsilyl- trifluoroacetamide) and MTBSTFA (N-methyl-N- tert-butyl- dimethyl-silyl trifluoroacetamide) were obtained from Sigma-Aldrich whereas streptozotocin (STZ) was bought from Bio Shop Canada. Invitrogen’s Pure Link RNA Mini Kit (Catalogue #12183018A) was manufactured by Thermo Fisher as well as Fisher Scientific’s Revert Aid First Strand cDNA Synthesis Kit (Catalogue #K1622). SYBR Select Master Mix (Catalogue No. 4472903) was used to carry out real-time expression of the target gene. All other reagents were of analytical grade.

### 2.4. Phytochemical Analysis of Fagonia cretica Using High-Performance Liquid Chromatography (HPLC)

The major compounds present in *F. cretica* aerial part’s methanol extract and its aqueous fraction were identified using the high-performance liquid chromatography (HPLC) analytical technique in gradient mode. The procedure was executed using HPLC Shimadzu Europe’s model SPD-10AV with pump LC-10AT. A sample of methanol extract and its aqueous fraction (5 mg/1 mL) was prepared in methanol. The samples were filtered using a 0.22 µm syringe filter before being injected into an HPLC column at 30 °C. The analysis was carried out at a flow rate of 1mL/min using 20% acetonitrile: 60% water: 20% methanol as mobile phase. The detection of compounds was carried out on a UV-visible detector at λ max 280 nm. The retention time at which the sample gave a peak was measured, and results were reported in ppm [26].

### 2.5. In Vivo Investigation of the Modulatory Effect of Fagonia cretica Extracts

#### 2.5.1. Experimental Animals

From the animal house of the University College of Pharmacy, University of the Punjab, Lahore, Sprague Dawley (SD) rats weighing 120–150 g were collected, which were 3–4 weeks old. Animals were kept at a constant temperature of (24 ± 1) °C, humidity (55–65%), and a 12 h light/dark cycle, with free access to feed and water ad libitum. Animals were acclimated to laboratory conditions for at least one week before their utilization in research. The Animal Ethical Committee of the University College of Pharmacy, University of the Punjab for the care and use of laboratory animals approved all protocols used in the PhD study vide letter no. D/271/FIMS.

#### 2.5.2. Induction and Maintenance of Diabetes

Chemically induced diabetes in laboratory animals can alter the enzymatic activity and expression of drug-metabolizing enzymes. Diabetes was induced in overnight-fasted rats by administering STZ intraperitoneally (*i.p*) (35 mg/kg) [4] dissolved in ice-cooled normal saline immediately before administration. Animals were only considered diabetic if their fasting blood glucose levels exceeded 200 mg/dL. Rats were fed with a high-fat diet (HFD) (unsalted butter) to provide 60% of calories from fat for the maintenance of rats in the diabetic state during the extract dose [27].

#### 2.5.3. Experimental Design

After acclimatization, rats were randomly assigned to two experimental groups, with 48 rats in each group. The rats were divided in first and second groups for the two experiments where each group was further divided into eight subgroups. These subgroups included: the control group that received the vehicle only and the diabetic control where the animals were *i.p.* administered with STZ (35 mg/kg) and observed for 10 days for the development of diabetes along with hepatic enzyme induction or inhibition. In the first experiment, the standard group for CYP3A4 was orally administered with ketoconazole (10 mg/kg) daily from the 11th day to the 20th day while the standard control for UGT2B7 was orally administered with Rifampicin (100 mg/kg) daily from the 11th day to the 20th day. However, the five treated groups in both experiments were orally administered with 500 mg/Kg methanol, aqueous, ethyl-acetate, chloroform, and *n*-hexane extracts through oral gavage daily from the 11th to the 20th day along with HFD. On the twentieth day of the sub-acute study, 1 h after the last dose of extracts and standards, each rat in all sub-groups of the first experiment (CYP3A4) was *i.p.* given midazolam (2 mg/kg) [28] while each rat in all sub-groups of the second experiment (UGT2B7) was *i.p.* administered with morphine (10 mg/kg) [29]. After 2 h of midazolam and morphine injection, all animals were anaesthetized by chloroform and sacrificed by abdominal venesection to obtain their liver. The liver was then stored at −80 °C until further analysis.

#### 2.5.4. Homogenization of Liver

The liver tissue was washed with ice-cold homogenizing buffer (0.01 M potassium phosphate buffer containing 1.15% potassium chloride) to remove blood. The liver tissue was dried and chopped into small pieces with surgical scissors before being weighed. To prepare liver homogenate, each gram of tissue received a five-fold volume in milliliters of ice-cold homogenizing buffer in a tissue homogenizer at 500–800 rpm [30] and stored at −80 °C.

#### 2.5.5. Isolation of mRNA and Real-Time PCR Quantitative Assay

Quantitative RT-PCR was used to evaluate the fold-change mRNA expression of target genes in the liver by using Applied Bio-systems Thermo-Scientific, Quant Studio-3, model #A28132. RNA extraction was performed according to the manufacturer’s instructions using Invitrogen’s Pure Link RNA Mini Kit and TRIzol reagent [31,32]. The relevant cycle thresholds (Cts) of samples were compared to controls and control samples containing house-keeping genes (GAPDH). The Ct values were normalized to those of GAPDH for all runs, and relative mRNA levels were quantified using the comparative cycle threshold method (ΔΔCts) [33]. The sequences of primers used in this study were GCCTGGTGCTCCTCTATCTA and GGCTGTTGACCATCATAAAAG for forward and reverse primers for CYP3A4 genes, respectively [34], whereas GGCCGATGGACTTTAGTCAC and CGTGTCCCATTCCTTGTGTC were the sequence of forward and reverse primers for amplification of rat UGT2B7 [35], respectively; meanwhile, AGTGCCAGCCTCGTCTCATA and ACCAGCTTCCCATTCTCAGC were GAPDH forward and reverse primers for its amplification of rat, respectively.

### 2.6. Sample Prepration for Detection and Quantitaion of CYP3A4 and UGT Substrates 

#### 2.6.1. Sample Preparation for Detection of CYP3A4 and UGT Substrates by ELISA

The sample preparation for the detection of CYP3A4 and UGT2B7 substrates in liver homogenates was performed according to the manufacturer’s instructions. The phosphate buffer was mixed with the liver homogenate, negative, positive, and high-positive controls for dilution. Negative, positive, and high positive controls were pipetted in duplicate into the first six wells of two microtiter plates, one for CYP3A4 substrate detection and the other for UGT2B7 substrate detection. Twenty µL of liver homogenate was manually pipetted into the next wells of two microtiter plates from all rats in both experimental groups. A drug-enzyme conjugate was added to microtiter plate wells and incubated for 45 min at room temperature. After incubation, the microtiter plates were washed five times with phosphate buffer for the removal of unbound drug–enzyme conjugates. The K-blue substrate (tetramethyl-benzidine plus hydrogen peroxide) was added to the plate wells and incubated for a further 30 min. After incubation, the reaction was stopped by adding a stop solution (a non-acid peroxidase acid solution) to plate wells with a yellow colorimetric end point. The microtiter plates were read at 450 nm using a multi scan sky-high microplate spectrophotometer from Thermo Fisher Scientific catalog # A51119500C, having a wavelength range of 200–1000 nm [36].

#### 2.6.2. Liquid–Liquid Extraction for CYP3A4 Substrate Quantitation by GCMS 

The extraction of midazolam and *alpha*-hydroxyl midazolam for quantitation in the liver homogenates was carried out by using the standard reported method of the liquid–liquid extraction technique. After derivatization, the solution was transferred into a GC vial, which was then placed on an auto-sampler for injection into the GC column [37]. Along with the extracted liver samples of rats in various groups, quality control and calibrators were also run on GCMS using the appropriate SIM mode for the quantitation of CYP3A4 substrate. The ions selected for quantitation in MSD were: *m*/*z* 345 and 347 for des-alkyl flurazepam; *m*/*z* 310, 312, and 325 for midazolam; and *m*/*z* 398, 400, and 399 for alpha hydroxyl midazolam.

#### 2.6.3. Solid–Liquid Extraction for UGT2B7 Substrate Quantitation by GCMS

The solid–liquid extraction (SPE) tubes were used for the extraction of morphine from liver homogenate by using the previously reported method [36]. The solution was transferred into a vial with a cap after derivatization and the vials were placed on an auto-sampler for injection into the GCMS. Gas chromatography–mass spectrometry (GCMS) used for analysis consists of an Agilent gas chromatograph model 6890N with a mass-selective detector (MSD) model 5973N and an injector model 7683. The separation of targeted molecules was carried out using a capillary column of length 30 m, an internal diameter of 0.25 mm, and a film thickness of 0.25 µm along with helium gas in a constant flow rate mode. Electron impact ionization and a selected ion-monitoring mode (SIM) were used in MSD. The ions selected for quantitation in MSD were: *m*/*z* 455 and 414 for nalorphine; and *m*/*z* 429, 236, and 287 for morphine. When mass spectrometry was used in SIM mode to quantify and identify an analyte of interest, the retention time match was necessary in addition to the usage of one quantifying ion, along with two qualifying ions.

### 2.7. Preparation of Working Solutions

Temazepam and morphine (50 and 100 ng/mL) were used as the positive control (PC) and high positive control (HPC), respectively, for detecting CYP3A4 and UGT2B7 substrates in the ELISA results interpretation. Des-alkyl flurazepam and nalorphine were used as the internal standards for quantitation of CYP3A4 and UGT2B7 substrates, having a strength of (2 mg/L) and (5000 ng/mL), respectively. Working positive quality control solutions Q1, Q2, and Q3 (100, 300, and 500 ng/mL) and calibrator working solutions C1, C2, C3, C4, and C5 (50, 100, 200, 400, and 600 ng/mL) containing midazolam and *alpha*-hydroxyl midazolam were prepared. Working positive quality control solutions Q1, Q2, and Q3 (40, 200, and 400 ng/mL) and calibrator working solutions C1, C2, C3, C4, C5, and C6 (10, 40, 100, 200, 400, and 600 ng/mL) containing morphine were prepared.

### 2.8. Method Validation

Method validation is an important step before the routine use of a specific method for the quantitation of drugs. Calibrators and quality control prepared in blank liver samples were used to validate the method. The following are the key steps involved in method validation.

#### 2.8.1. Specificity

The method specificity was determined by analyzing at least six blank liver samples. No interference from the liver matrix was observed at the specific retention time window of the internal standard and targeted molecule [38].

#### 2.8.2. Construction of Calibration Curve and Linearity

The linearity of the method was developed by spiking the following concentrations of midazolam and *alpha*-hydroxyl midazolam: 50, 100, 200, 400 and 600 ng/mL, and for morphine, 10, 40, 100, 200, 400 and 600 ng/mL in negative liver samples and running all calibrator levels in triplicate. The r^2^ coefficient of determination of ≥0.985 will be acceptable [38].

#### 2.8.3. Sensitivity

The limit of detection (LOD) and lower limit of quantitation (LLOQ) were used to develop the sensitivity of the method. The LOD determined using this method for midazolam and *alpha*-hydroxyl midazolam was 50 ng/mL and for morphine was 20 ng/mL. The LLOQ of the procedure that was found with the greatest degree of accuracy and precision was 100 ng/mL for midazolam and *alpha*-hydroxyl midazolam and 40 ng/mL for morphine [38].

#### 2.8.4. Accuracy and Precision

The accuracy and precision of the method were developed by using three different concentrations (QC1, QC2, and QC3) of midazolam and *alpha*-hydroxyl midazolam: 100, 300, and 500 ng/mL, and morphine: 40, 200, and 400 ng/mL. A single sample with three different concentrations was run for three separate days inter-day (reproducibility), and three different concentrations were run three times in a single day/intra-day (repeatability) [38]. The acceptance criteria for quantification of an analyte of interest should be 20% relative to that of averaged calibrators or controls run during GCMS analysis.

### 2.9. Analysis Method for the Detection of CYP3A4 and UGT2B7 Substrates by ELISA

ELISA is a semi-quantitative method for detecting analytes of interest in biological specimens, and it was used to detect CYP3A4 and UGT2B7 substrates in liver homogenates. The ELISA results were interpreted using the following equation: NC > PC > HPC. The absorbance value of negative control NC should be greater than that of PC and HPC, whereas the absorbance value of PC should be greater than that of HPC. Since an immunoassay is a screening technique, all presumptively positive samples of liver homogenates were confirmed using a second technique which uses a different chemical principle, such as gas or liquid chromatography with mass spectrometry [39]. For this reason, further analysis was performed on GC/MS in SIM mode in order to determine the exact concentration of CYP3A4 and UGT2B7 substrates metabolized.

### 2.10. Analysis Method for the Quantitation of Alpha-Hydroxyl Midazolam and Morphine by GCMS

After extraction and derivatization, homogenized liver from all experimental groups was tested using GC/MS in SIM mode to measure the concentration of *alpha*-hydroxyl midazolam, and metabolized morphine. Enhanced data analysis software from Agilent Technology was used to create the calibration curve. Using the internal standard technique, the software performed quantitative calculations for calibrators, controls, and liver homogenates. The concentration of *alpha*-hydroxyl midazolam and morphine metabolized was measured by comparing the unknown ratios, obtained from homogenized liver to standard ratios.

### 2.11. Molecular Docking Study

Molecular docking studies were performed for the main detected metabolites present in *F. cretica* methanol extract and its aqueous fraction within the active sites of CYP3A4 (PDB code: 4D7D; 2.76 Å) [40] that was downloaded from protein data bank. This was achieved by Discovery Studio 4.5 (Accelrys Inc., San Diego, CA, USA) adopting the C-Docker protocol in which the binding energies (∆G) were estimated from the equation as previously reported [41,42,43,44].

### 2.12. Statistical Analysis

Graph pad prism version 5.0 was used for statistical analysis. The results were presented as mean ± SEM. The data were analyzed using a one-way analysis of variance (ANOVA). A Tukey test was applied to examine significance among the groups. (*p* < 0.05) was considered significant.

## 3. Results

### 3.1. Phytochemical Analysis of Fagonia cretica Using High-Performance Liquid Chromatography (HPLC)

**Figure 1:**
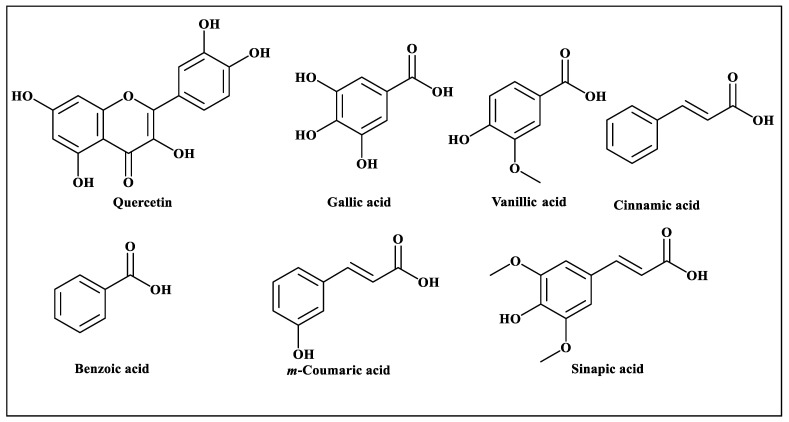
Scheme showing the chemical structures of the identified compounds in the methanol and aqueous extracts of *F. cretica* using high-performance liquid chromatography (HPLC).

Methanol and aqueous fraction were chosen for further investigations as the reported literature review of *F. cretica* revealed that methanol, ethanol, and aqueous extracts showed pharmacological activities of the plant. Furthermore, the phytochemical investigation of *F. cretica* extracts reported in the literature also revealed the presence of active phytochemicals, mainly flavonoids and phenolic compounds, in methanol, ethanol and aqueous extracts [22]. Moreover, a recent study has also shown that non-polar extracts of *F. cretica* lack of active known phytochemicals [45]. The HPLC analysis of *F. cretica* methanol extract and its aqueous fraction provided quantitative data on flavonoids and phenolic compounds. The methanol extract contained more flavonoids and phenolic compounds in comparison to the aqueous fraction of *F. cretica*. These phenolic compounds include quercetin, gallic acid, vanillic acid, benzoic acid, *m*-coumaric acid, cinnamic acid and sinapic acid (Table 1). A scheme showing the chemical structures of the identified compounds is illustrated in Figure 1.

### 3.2. GC/MS Method Validation

**Figure 2:**
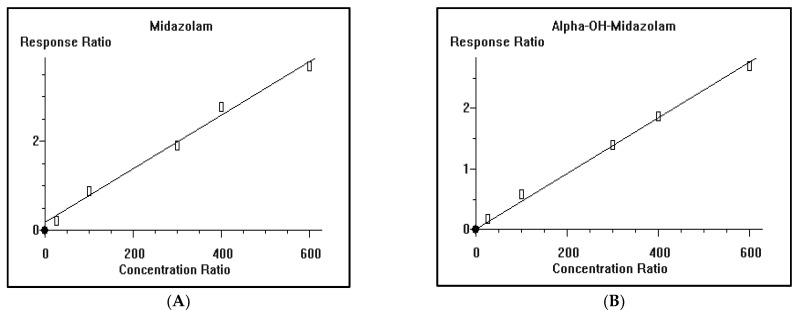
Representative calibration curves of midazolam (**A**) and *alpha*-hydroxyl midazolam (**B**) with a coefficient of determination (r^2^) = 0.990066 and 0.998683, respectively. Cal-1, Cal-2, Cal-3, Cal-4, and Cal-5 calibrators, having a concentration of midazolam and *alpha*-hydroxyl midazolam of 50, 100, 200, 400, and 600 ng/mL, respectively, were used to develop the calibration curve.

The calibration curves of midazolam (A) and *alpha*-hydroxyl midazolam (B) were constructed where Cal-1, Cal-2, Cal-3, Cal-4, and Cal-5 calibrators, having a concentration of midazolam and *alpha*-hydroxyl midazolam of 50, 100, 200, 400, and 600 ng/mL, respectively, were used to develop the calibration curve. The calibration curves of midazolam (A) and *alpha*-hydroxyl midazolam (B) showed a coefficient of determination (r^2^) = 0.990066 and 0.998683, respectively (Figure 2).

**Figure 3:**
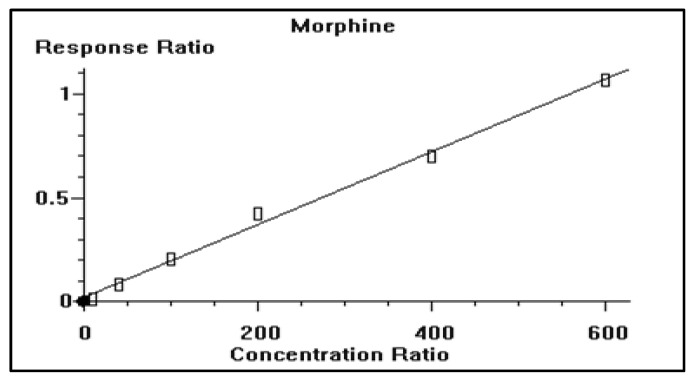
Representative calibration curve of morphine with a coefficient of determination (r^2^) = 0.990066 and 0.995186. Cal-1, Cal-2, Cal-3, Cal-4, Cal-5 and Cal-6 calibrators with morphine concentration of 10, 40, 100, 200, 400, and 600 ng/mL used to develop the calibration curve.

Meanwhile, the calibration curve of morphine was constructed in which Cal-1, Cal-2, Cal-3, Cal-4, Cal-5, and Cal-6 calibrators, with morphine concentrations of 10, 40, 100, 200, 400, and 600 ng/mL, were used to develop the calibration curve. The calibration curves of morphine showed a coefficient of determination (r^2^) of 0.995186 (Figure 3).

The spiked concentration of quality control samples obtained from the calibration curves showed the 20% midazolam, *alpha*-hydroxy midazolam, and morphine target concentration in the quality control samples. Accuracy (%Bias) and precision (%CV) were measured, and the acceptable ranges for accuracy and precision were ±20% at each concentration. The method fits the quantitative analysis of analytes of interest as illustrated in Table 2.

### 3.3. Effect of F. cretica Extracts on the Activity and mRNA Expression of CYP3A4 and UGT2B7

#### 3.3.1. Effect of *F. cretica* Extracts on the Activity of CYP3A and UGT2B7 by ELISA

The effects of *F. cretica* extracts on the metabolism of midazolam and morphine by drug-metabolizing enzymes were initially assessed by using an ELISA screening technique having adequate sensitivity for detection. The absorbance values of liver homogenate in both experimental groups were less than or equal to those of the positive control (PC), indicating the presence of CYP3A4 and UGT2B7 substrates in liver homogenates.

#### 3.3.2. Effect of *F. cretica* Extracts on the Activity of CYP3A4 by GCMS

**Figure 4:**
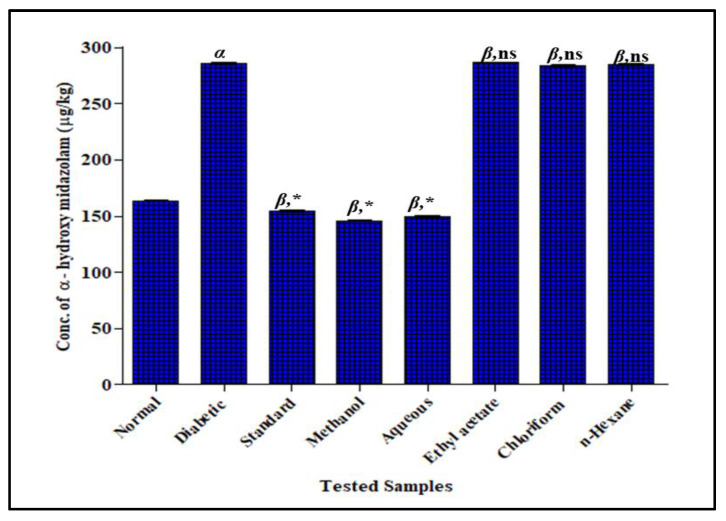
Effect of *F. cretica* extracts on activity of CYP3A4 in diabetic rats. The results were analyzed by applying one-way ANOVA followed by Tukey’s test and are expressed as Mean ± SEM; (*n* = 6). The comparison of the diabetic group with the normal group is presented as “α”. Ketoconazole and extract treatment groups’ comparisons with the diabetic group are presented as “β” on graph bars. * *p* < 0.05 was considered significant while ^ns^ *p* > 0.05 was considered non-significant.

In STZ-induced diabetic rats, the concentration of *alpha*-hydroxyl midazolam was increased in a significant way by 285.8 ± 0.82 mg/Kg when STZ was administered at a single dose of 35 mg/kg as compared to normal rats 163.8 ± 0.81 mg/Kg. The concentration of *alpha*-hydroxyl midazolam was decreased significantly when rats were treated with *F. cretica* extracts after diabetes induction. The methanol extract and its aqueous fraction presented the most significant potential to decrease the concentration of *alpha*-hydroxyl midazolam with a mean value of 176.0 ± 0.85 mg/Kg and 182.9 ± 0.99 mg/Kg, respectively, when compared with the concentration of *alpha*-hydroxyl midazolam in the STZ-induced diabetic group. The concentration of *alpha*-hydroxyl midazolam in methanol extract and its aqueous fraction groups was comparable to the ketoconazole group that showed a 154.6 ± 0.90 mg/Kg reduction with respect to STZ-induced groups, verifying that the inhibitory potential of *F. cretica* on CYP3A4 metabolism was as effective as ketoconazole’s inhibitory potential. The ethyl-acetate, chloroform, and *n*-hexane extracts of *F. cretica* had no effect on midazolam metabolism when compared to the diabetic group; the results are displayed in Figure 4.

#### 3.3.3. Effect of *F. cretica* Extracts on Fold-Change mRNA Expression of CYP3A4 by PCR

**Figure 5:**
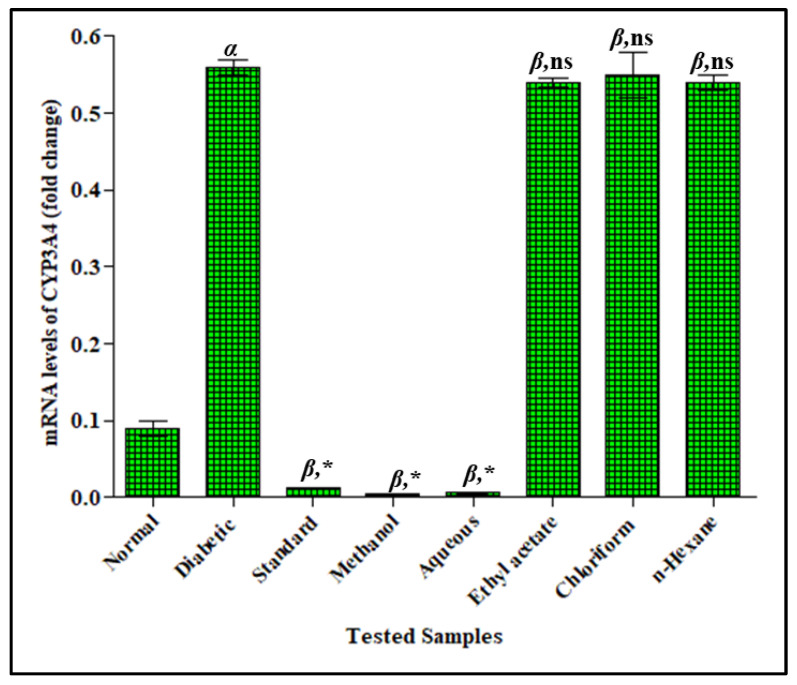
Effect of *F. cretica* extracts on fold-change mRNA levels of CYP3A4 in diabetic rats. The results were analyzed by applying one-way ANOVA followed by Tukey’s test and are expressed as Mean ± SEM; (*n* = 6). The comparison of the diabetic group with the normal group is presented as “α”. Ketoconazole and treatment groups’ comparisons with the diabetic group are presented as “β” on graph bars. * *p* < 0.05 was considered significant while ^ns^ *p* > 0.05 was considered non-significant.

The fold-change mRNA expression of CYP3A4 were significantly increased in the STZ-induced diabetic rats by 0.56 ± 0.01 when compared to the control group 0.09 ± 0.01. The increased fold-change mRNA expression of CYP3A was decreased in a significant way when *F. cretica* extract treatments were given to diabetic rats. The methanol extract and its aqueous fraction of *F. cretica* showed the most significant effects by 0.15 ± 0.002 and 0.16 ± 0.001, respectively, when compared with the diabetic group. The inhibitory potential of methanol extract and its aqueous fraction of *F. cretica* on the fold-change mRNA expression of CYP3A4 was significantly comparable to that of the standard CYP3A4 inhibitor ketoconazole that showed 0.013 ± 0.002 with respect to the diabetic group. The ethyl-acetate, chloroform, and *n*-hexane extracts of *F. cretica* did not affect the mRNA expression of CYP3A4 in diabetic rats; the results are shown in Figure 5.

#### 3.3.4. Effect of *F. cretica* Extracts on the Activity of UGT2B7 by GCMS

In STZ-induced diabetic rats, the concentration of un-metabolized morphine was increased in a significant way by 251.2 ± 0.99 mg/Kg when STZ was administered at a single dose of 35 mg/kg as compared to normal rats 82.0 ± 0.92 mg/Kg. The un-metabolized morphine concentration was significantly decreased in rats treated with *F. cretica* extracts after diabetes induction. The methanol extract and its aqueous fraction showed the most significant induction potential to decrease the concentration of un-metabolized morphine, displaying 93.4 ± 0.96 mg/Kg and 96.4 ± 1.27 mg/Kg, respectively, when compared with the un-metabolized concentration of morphine in the diabetic group. The induction potential of methanol extract and the aqueous fraction of *F. cretica* was significantly comparable to that of the standard rifampicin group that showed a 64.8 ± 0.99 mg/Kg reduction with respect to the diabetic group.

**Figure 6:**
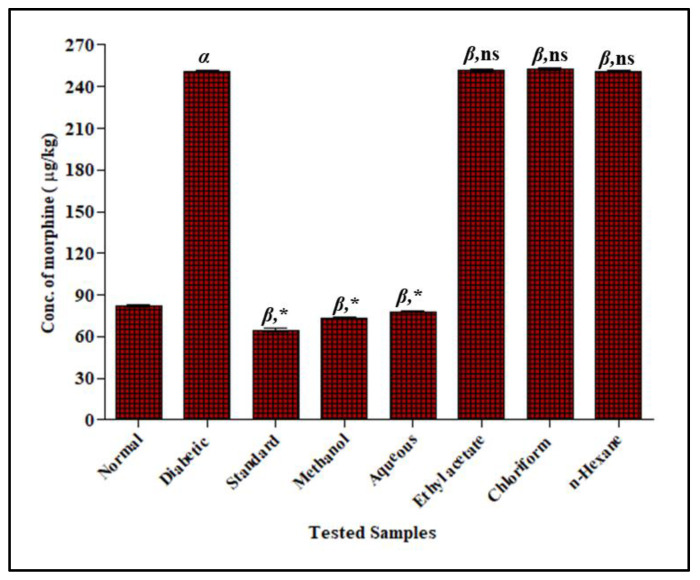
Effect of *F. cretica* extracts on activity of UGT2B7 in diabetic rats. The results were analyzed by applying one-way ANOVA followed by Tukey’s test and are expressed as Mean ± SEM; (*n* = 6). The comparison of the diabetic group with the normal group is presented as “α”. Rifampicin and extract treatment groups’ comparisons with the diabetic group are presented as “β” on graph bars. * *p* < 0.05 was considered significant while ^ns^ *p* > 0.05 was considered non-significant.

The induction potential of *F. cretica* was as effective as the rifampicin induction potential on the activity of UGT2B7. The administration of ethyl-acetate, chloroform, and *n*-hexane extracts of *F. cretica* to diabetic rats showed a non-significant effect on the morphine metabolism as illustrated in Figure 6.

#### 3.3.5. Effect of *F. cretica* Extracts on Fold-Change mRNA Expression of UGT2B7 by PCR

**Figure 7:**
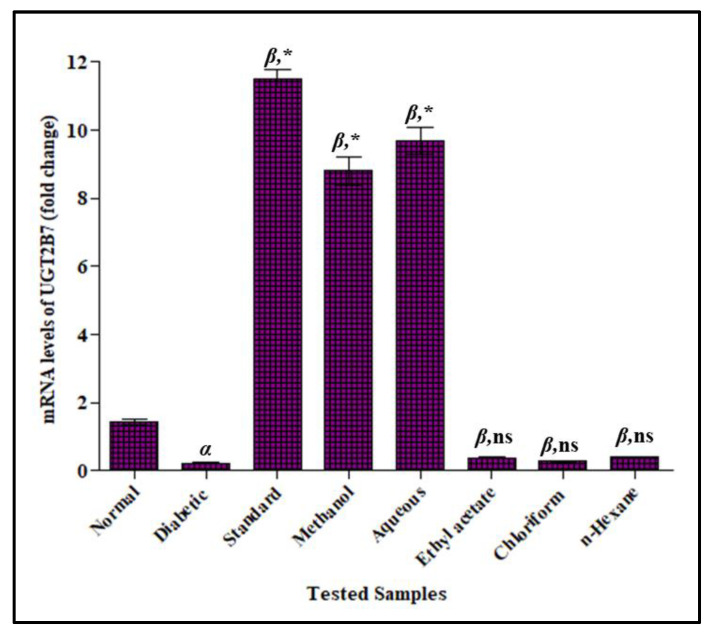
Effect of *F. cretica* extracts on fold-change mRNA levels of UGT2B7 in diabetic rats. The results were analyzed by applying one-way ANOVA followed by Tukey’s test and are expressed as Mean ± SEM; (*n* = 6). The comparison of the diabetic group with the normal group is presented as “α”. Rifampicin and extract treatment groups’ comparisons with the diabetic group are presented as “β” on graph bars. * *p* < 0.05 was considered significant while ^ns^ *p* > 0.05 was considered non-significant.

The fold-change mRNA expression of UGT2B7 were significantly lowered in the STZ-induced diabetic rats by 0.23 ± 0.02 as compared to the normal group 9.18 ± 0.39. When the diabetic rats were given *F. cretica* extract treatments, their mRNA expression of UGT2B7 were significantly increased. When compared to the diabetic group, methanol extract and the aqueous fraction of *F. cretica* showed the most significant increasing effects, estimated by 8.14 ± 0.26 and 7.17 ± 0.23 respectively. The induction potential of the plant on the mRNA levels of UGT2B7 was comparable to that of the standard inducer, rifampicin that showed 11.52 ± 0.25 induction as compared to diabetic rats. The fold-change mRNA expression of UGT2B7 in diabetic rats were not significantly altered by ethyl acetate, chloroform, and *n*-hexane extracts of *F. cretica* as shown in Figure 7.

### 3.4. Molecular Docking Study

**Figure 8:**
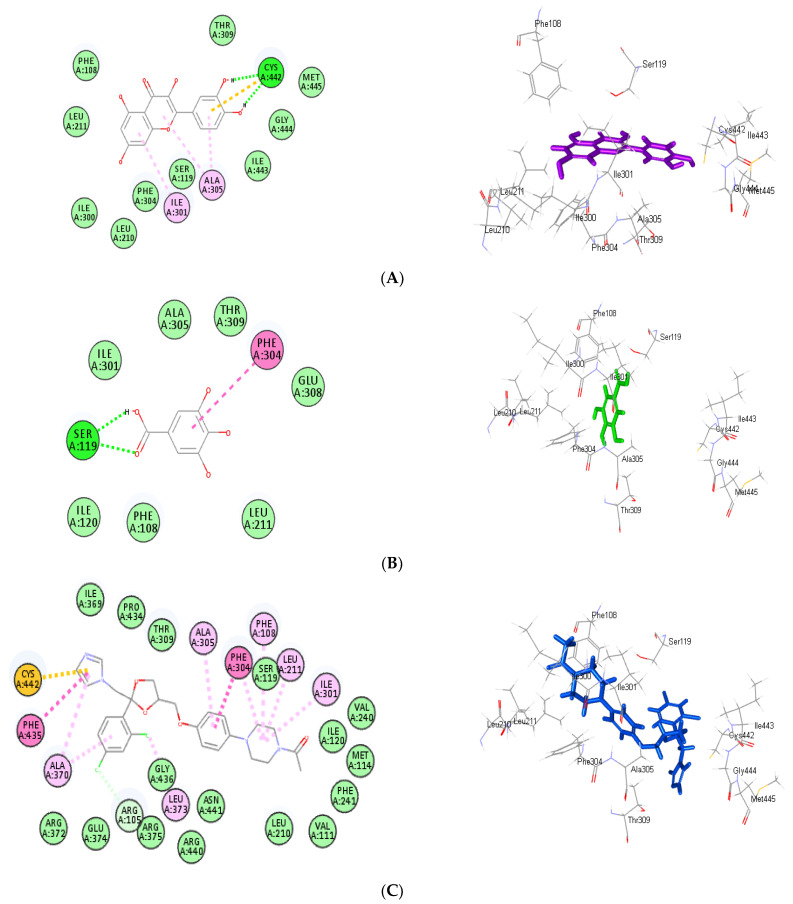
Two-dimensional and three-dimensional binding modes of quercetin (**A**), gallic acid (**B**) and ketoconazole (**C**) within the active center of (CYP3A) (PDB code: (4D7D) using in silico studies).

Molecular docking was performed for the main detected metabolites present in *F. cretica* methanol extract and its aqueous fraction within the active sites of CYP3A4. All the examined compounds showed certain inhibition *versus* CYP3A4 active sites (Table 3). Quercetin showed the best fitting within the active pocket followed by gallic acid displaying free binding energies (∆G) of −30.83 and −23.12 kcal/mol, respectively, approaching in this regard ketoconazole that showed ∆G of −30.16 kcal/mol; meanwhile, CYP3A4 co-crystalized ligand, PKT: tert-butyl [(2S)-1-(1H-indol-3-yl)-3-({3-oxo-3- [(pyridin-3-ylmethyl)amino]propyl}sulfanyl)propan-2-yl]carbamate, displayed ∆G of −46.88 kcal/mol. Quercetin exhibited two conventional H-bonds with Cys442 in addition to three π-alkyl bonds with Ala305, Ile301 and one π-sulfur bond with Cys442 amino acid residues at the active sites (Figure 8A). Regarding gallic acid, it formed two H-bonds with Ser119 and one π-π bond with Phe304 in addition to Van der Waals interactions with amino acid residues at the active site (Figure 8B). Concerning ketoconazole, it exerted two π-π bonds with Phe435 and Phe304; one π-sulfur bond with Cys442; seven alkyl and π-alkyl bonds with Ala370, Phe108, Leu211, Ala305, and Ile301; and one C-H bond with Arg105 (Figure 8C). However, the illustrated results are in accordance with what was previously reported where quercetin was previously reported to inhibit CYP3A4 enzyme activity in vitro in a concentration-dependent manner with a 50% inhibition concentration (IC_50_) of 1.97 μM [46]. Furthermore, gallic acid is a principal polyphenol in many fruits and plants; its inhibitory potential on CYP3A4 was previously reported where it triggered a concentration-dependent loss of CYP3A4 activity, with IC_50_ values of 615.2 μM and 669.5 μM in human liver microsomes and recombinant CYP3A4 systems, respectively [47].

## 4. Discussion

In the present era, natural remedies are sought to cure and manage diseases such as hypertension, cancer, liver diseases, and diabetes mellitus. Diabetes mellitus is characterized by complex metabolic disorders that cause changes in metabolism as well as the therapeutic effects and risks of drugs used by diabetic patients [48]. Diabetes mellitus significantly alters the pharmacokinetics of cardiovascular, antihypertensive, and lipid-lowering drugs [49]. The anti-diabetic potential of *F. cretica* suggested its beneficial effect in the amelioration of diabetes mellitus [50]. Monitoring the variations in CYP3A4 and UGT2B7 activity in diabetic patients is crucial for minimizing toxicity caused by drug–drug and drug–disease pharmacokinetic interactions. The primary goal of the current study is to determine the effect of various extracts of *F. cretica* on these drug-metabolizing enzymes in streptozotocin-induced diabetic rats.

The short-acting midazolam is commonly used in vivo as a probe for the determination of CYP3A4 activity. Midazolam was hydroxylated by CYP3A4 to form the major metabolite *alpha*-hydroxyl midazolam, as potent as the parent drug, and the minor metabolite 4-hydroxyl midazolam [51]. Morphine, after oral administration, was completely absorbed from the gut [52] and transported to the liver for rapid metabolism into two main metabolites: morphine-3-glucuronide (M3G) and morphine-6-glucuronide (M6G) [53]. The co-administration of the usual therapeutic doses of ketoconazole increased the area under the plasma concentration curve of midazolam and triazolam by 10 fold or more [54] as it acts as a highly potent CYP3A4 inhibitor with an inhibition constant (Ki) value in the nanomolar range [55]. Meanwhile, the area under the curve (AUC) of morphine, M3G, and M6G decreased after rifampicin exposure; the pain thresholds were equivalent to placebo and morphine glucuronide urine recovery was significantly reduced [56]. Morphine analgesic benefits were reduced after 13 days of rifampicin therapy, and the area under the curves of morphine, M3G, and M6G were reduced by 28% [29].

Furthermore, studies revealed the evidence of hepatic CYP3A4 induction in diabetes. In diabetic rats, CYP3A4 induction caused a faster metabolism and a lower area-under-the-curve value for clarithromycin and telithromycin [57]. Similar induction in CYP3A4 was reported in the current study with midazolam which was considered as a true substrate for the activity of CYP3A4. In the present study, the effect of *F. cretica* on the increased activity of CYP3A4 caused by mRNA overexpression in diabetic rats was investigated. In streptozotocin-induced diabetic rats, CYP3A4 induction increased the systemic clearance of midazolam and reduced its therapeutic effects. However, methanol extract and its aqueous fraction of *F. cretica* (500 mg/kg) exhibited a significant inhibitory potential in the attenuation of increased activity of CYP3A4.

They showed significant inhibitory potential against CYP3A4 when compared to the standard drug control ketoconazole. PXR regulates CYP3A4 expression, and CYP3A4 induction was linked to the interaction of a relevant chemical or ligand with a pregnane X receptor [58]. PXR was reported to have an effective regulatory impact on the expression of UGT2B7 protein [59]. There is a positive relation between PXR and UGT2B7 mRNA in human liver microsomal protein. UGT2B7 often has been considered a gene responsive to PXR, as indicated by the ability of rifampin, a PXR ligand, to induce expression [60]. PXR can be activated by a variety of xenobiotics and the activation of PXR leads to the regulation of phase I and phase II enzymes and drug transporters [61]. PXR regulates the expression of CYP3A4 both naturally and when it is stimulated. PXR activation is the underlying reason for numerous pharmacological interactions involving modifications in CYP3A4 expression, making it a high affinity site for many drug agonists [62]. The most significant PXR substrates are members of the CYP 3A enzyme family that is involved in the metabolism of more than 50% of drugs [63]. The inhibitory potential of *F. cretica* may be linked with its ability to interact with the PXR pathway which works through decreasing PXR activation leading to reduced de novo synthesis of CYP3A4 enzyme. This may be linked to the richness of these extracts with flavonoids and phenolic compounds. Flavonoids and phenolic compounds were reported to exhibit significant potential in the management of interactions due to their inhibitory and inducing activity on drug-metabolizing enzymes [64] and this was consolidated via molecular docking studies where all the identified flavonoid and phenolic acid identified in methanol extract and its aqueous fraction revealed certain inhibition to CYP3A4, particularly quercetin and gallic acid that showed an inhibitory potential approaching that of ketoconazole. However, the ethyl-acetate, chloroform, and *n*-hexane extracts of *F. cretica* had no effect on the induction and expression of CYP3A4 in diabetic rats.

Moreover, diabetes mellitus also reduces the enzymatic activity and expression of phase II metabolizing enzyme UGT2B7 [65]. In the current study, UGT2B7 was found to be similarly inhibited leading to a decrease in the morphine metabolism, a validated substrate for assessing UGT2B7 activity [66]. The study revealed that *F. cretica * extracts exhibited significant inductive potential in the amelioration of inhibited activity of UGT2B7 in diabetic rats. The concentration of un-metabolized morphine in the current study was significantly increased due to the inhibition of the activity of UGT2B7 in diabetic rats. Both methanol extract and its aqueous fraction of *F. cretica* (500 mg/kg) had a significant potential to decrease the concentration of un-metabolized morphine. Studies have revealed the evidence that mRNA expression of UGT2B7 was down-regulated in diabetes [35]. When compared to the control group, the mRNA expression of UGT2B7 was significantly inhibited in the STZ-induced diabetic rats. However, when diabetic rats were treated with methanol and aqueous extract, the mRNA expression of UGT2B7 was significantly (*p* < 0.05) increased. The concentration of un-metabolized UGT2B7 substrate and mRNA expression were likewise comparable to the standard UGT2B7 inducer rifampicin, which is known to induce UGT2B7 activity via the activation of the PXR pathway for enzyme induction [60]. The mechanism underlying methanol and its aqueous fraction induction potential could be related to its ability to activate the PXR pathway, which leads to increased de novo UGT2B7 enzyme synthesis in a manner similar to rifampicin. It is proposed that phytochemical components of the plant activate PXR and significantly improve UGT2B7 under-expression in diabetic rats via a PXR-mediated mechanism. The activity and mRNA expression of UGT2B7 were not significantly modulated in rats treated with *n*-hexane, chloroform, and ethyl-acetate extracts of *F.cretica* after diabetes induction, implying that these extracts had no potential to modulate drug pharmacokinetics.

The study showed that flavonoids and phenolic compounds significantly regulate the activity and expression of drug-metabolizing enzymes via modulating PXR-mediated mechanisms. It is thought that *F. cretica’s* inhibitory and induction potential are due to the abundance of flavonoids and phenolic compounds, which may work through PXR-mediated pathways, resulting in an increase and decrease in enzyme de novo protein synthesis. The liver is the key site of drug metabolism; therefore, reduced activity and expression of CYP3A4 may enhance the therapeutic effects of CYP3A4 substrates in diabetes mellitus. Similarly, overexpression of UGT2B7 mRNA increases UGT2B7 activity, enhancing the efficacy of its substrates, hence lowering the likelihood of drug–drug or disease interactions.

*F. cretica* showed anti-diabetic and anti-cancer pharmacological activity that is a convincing argument for its usage in populations to take advantage of its protective capacity to alter the activity of phase-I and phase-II drug-metabolizing enzymes to prevent interactions. As a result, *F. cretica’s* anti-diabetic and anti-cancer potential, as well as its protective potential, implied that it could be administered as a supplement to conventional anti-diabetic and anti-cancer therapy to prevent pharmacokinetic drug–drug and drug–disease interactions.

## 5. Conclusions

From the foregoing study, it was concluded that *F. cretica* aerial parts, particularly the methanol extract and its aqueous fraction, showed a significant potential to reduce the possibility of drug–drug or disease interactions in diabetic rats by modulating the activity and mRNA expression of CYP3A4 and UGT2B7 drug-metabolizing enzymes. HPLC analysis of *F. cretica* aerial parts’ methanol extract and its aqueous fraction revealed their richness with flavonoids and phenolic compounds. The obtained biological activity was further consolidated with molecular docking studies where all the identified compounds revealed certain inhibition to CYP3A4, particularly quercetin and gallic acid that showed an inhibitory potential approaching that of ketoconazole. This suggested the use of *F. cretica* as a complementary medicine with standard anti-diabetic therapy. Further studies for the standardization of extracts, isolation, and the mechanistic elucidation of the active constituents of *F. cretica* are highly recommended.

## Figures and Tables

**Table 1:** Quantitative estimation of phytochemicals in the methanol and aqueous extracts of *Fagonia cretica* using high-performance liquid chromatography (HPLC).

Compounds Name	Methanol Extract		Aqueous Extract	
	Retention Time (min)	Amount (ppm)	Retention Time (min)	Amount (ppm)
Quercetin	2.707	8.28	2.727	7.82
Gallic acid	4.247	2.52	4.240	2.43
Vanillic acid	13.627	6.72	13.400	5.64
Benzoic acid	14.667	11.21	14.533	8.66
*m*-Coumaric acid	20.367	2.14	19.680	0.97
Cinnamic acid	25.040	4.25	-	-
Sinapic acid	26.013	2.01	26.453	1.64

**Table 2:** GCMS method validation data for midazolam, *alpha*-hydroxy midazolam, and morphine quantification.

Drug	Nominal Value	Day-1			Day-2			Day-3			Grand Average	STD	% CV	% Bias
		Run1	Run2	Run3	Run1	Run2	Run3	Run1	Run2	Run3				
Midazolam	100	112.5	91.8	105.7	100.8	97.8	93.4	111.7	103.9	101.5	102.1	7.24	7.09	2.12
	300	325.4	311.6	305.4	325.8	304.5	298.8	278.2	318.7	308.5	308.6	14.74	4.78	2.85
	500	517.2	467.8	506.8	496.7	546.9	508.9	510.2	481.7	555.9	510.2	28.04	5.50	2.05
*Alpha* Hydroxyl Midazolam	100	104.6	99.6	105.5	109.5	97.8	95.6	102.8	106.8	110.3	103.6	5.11	4.93	3.61
	300	291.2	301.5	316.5	308.6	278.9	292.7	310.8	322.7	333.4	306.3	17.03	5.56	2.08
	500	496.8	472.7	545.8	542.9	545.8	509.4	515.7	529.3	501.9	517.8	25.33	4.89	3.56
Morphine	40	36.3	39.2	41.8	37.2	43.7	41.0	44.1	40.4	46.2	41.1	3.25	7.91	2.75
	200	197.5	211.6	178.6	217.7	208.3	205.5	217.1	195.6	222.6	206.1	13.71	6.65	3.03
	400	439.0	417.9	388.2	391.7	412.9	444.7	369.8	433.0	420.2	413.0	25.23	6.11	3.26

The results are expressed as Mean ± STD. For each concentration of quality control sample, accuracy (% CV) and precision (% Bias) were measured within 20% acceptable ranges.

**Table 3:** Free binding energies (kcal/mol) of main detected metabolites present in *F. cretica* methanol extract and its aqueous fraction within the active sites of CYP3A4 using in silico studies.

Compound	CYP3A4 (4D7D)	Number of Formed Hydrogen Bonds and C-H Bonds	Number of Formed π-π and π-alkyl Bonds
Benzoic acid	−12.30	2; Ser119	1; Phe304
Cinnamic acid	−17.32	2; Cys442, Ile443	-
Gallic acid	−23.12	2; Ser119	1; Phe304
*m*-Coumaric_acid	−18.41	1; Cys442	1; Ala305
Quercetin	−30.83	2; Cys442	3; Ala305, Ile301
Sinapic acid	−22.47	5; Arg375, Arg105, Leu373, Phe435, Arg372	1; Ala370
Vanillic acid	−19.42	1; Glu308	-
Ketoconazole	−30.16	-	10; Phe435, Ala370, Phe108, Leu211, Ala305, Phe304, Ile301
Co-crystalized ligand	−46.88	1; Arg375	6; Leu, Ala370, Leu211, Phe304, Ile301, Phe108

## Data Availability

Data are available in the manuscript.
